# Effects of Acupuncture at GV20 and ST36 on the Expression of Matrix Metalloproteinase 2, Aquaporin 4, and Aquaporin 9 in Rats Subjected to Cerebral Ischemia/Reperfusion Injury

**DOI:** 10.1371/journal.pone.0097488

**Published:** 2014-05-14

**Authors:** Hong Xu, Yamin Zhang, Hua Sun, Suhui Chen, Fuming Wang

**Affiliations:** Department of Traditional Chinese Medicine, Peking Union Medical College Hospital (PUMCH), Peking Union Medical College (PUMC), Chinese Academy of Medical Sciences, Beijing, China; National University of Singapore, Singapore

## Abstract

**Background/Purpose:**

Ischemic stroke is characterized by high morbidity and mortality worldwide. Matrix metalloproteinase 2 (MMP2), aquaporin (AQP) 4, and AQP9 are linked to permeabilization of the blood-brain barrier (BBB) in cerebral ischemia/reperfusion injury (CIRI). BBB disruption, tissue inflammation, and MMP/AQP upregulation jointly provoke brain edema/swelling after CIRI, while acupuncture and electroacupuncture can alleviate CIRI symptoms. This study evaluated the hypothesis that acupuncture and electroacupuncture can similarly exert neuroprotective actions in a rat model of middle cerebral artery occlusion (MCAO) by modulating MMP2/AQP4/APQ9 expression and inflammatory cell infiltration.

**Methods:**

Eighty 8-week-old Sprague-Dawley rats were randomly divided into sham group S, MCAO model group M, acupuncture group A, electroacupuncture group EA, and edaravone group ED. The MCAO model was established by placement of a suture to block the middle carotid artery, and reperfusion was triggered by suture removal in all groups except group S. Acupuncture and electroacupuncture were administered at acupoints GV20 (governing vessel-20) and ST36 (stomach-36). Rats in groups A, EA, and ED received acupuncture, electroacupuncture, or edaravone, respectively, immediately after MCAO. Neurological function (assessed using the Modified Neurological Severity Score), infarct volume, MMP2/AQP4/AQP9 mRNA and protein expression, and inflammatory cell infiltration were all evaluated at 24 h post-reperfusion.

**Results:**

Acupuncture and electroacupuncture significantly decreased infarct size and improved neurological function. Furthermore, target mRNA and protein levels and inflammatory cell infiltration were significantly reduced in groups A, EA, and ED vs. group M. However, MMP2/AQP levels and inflammatory cell infiltration were generally higher in groups A and EA than in group ED except MMP2 mRNA levels.

**Conclusions:**

Acupuncture and electroacupuncture at GV20 and ST36 both exercised neuroprotective actions in a rat model of MCAO, with no clear differences between groups A and EA. Therefore, acupuncture and electroacupuncture might find utility as adjunctive and complementary treatments to supplement conventional therapy for ischemic stroke.

## Introduction

Ischemic stroke accounts for more than 80% of all stroke cases and has a high morbidity and mortality worldwide [1], [2]. Reperfusion damage occurs when blood returns to the brain after a period of ischemia, continuing even after blood flow is restored [3]. Accordingly, reperfusion occupies an important position in the pathophysiology of cerebral ischemia [4], and many pathological events are associated with cerebral ischemia/reperfusion injury (CIRI). These events encompass inflammation, increased production of reactive oxygen species, blood-brain barrier (BBB) disruption, brain edema, necrosis, and apoptosis.

Inflammation in CIRI is characterized by the rapid activation of resident microglia and the infiltration of inflammatory cells, including myeloperoxidase (MPO)^+^ neutrophils, cluster of differentiation (CD) 68^+^ monocytes/macrophages, and leukocytes. In the early stages of ischemic stroke (hours to days), proinflammatory mediators (e.g., cytokines and matrix metalloproteinases (MMPs)) are released by resident microglia and infiltrating cells [5]. Infiltrating leukocytes release interleukin-1β, tumor necrosis factor-α, and interleukin-6, and infiltrating macrophages and neutrophils join leukocytes to induce/activate MMPs. Cerebral inflammatory responses are then amplified by the actions of cytokines and MMPs, the disruption of the BBB, and the development of brain edema [5].

The BBB crucially contributes to brain homeostasis [6] and is mostly formed by the endothelial cells of the microvasculature. The BBB facilitates selective, diffusion-mediated exchange of membrane-permeant molecules between the circulating blood and the central nervous system, and in this manner protects the brain from extraneous compounds and neurotoxic substances. Microvessel endothelial cells are connected to each other, to surrounding pericytes, and to the foot processes of astrocytes by tight junctions. These cells work together to uphold normal BBB function.

The composition and structure of the BBB includes many factors that either maintain or disturb the fluid balance in the brain during normal and pathological processes. For example, astrocytes secrete the pro-ischemic mediator, transforming growth factor-β, during pathological processes such as CIRI; transforming growth factor-β then goes on to affect the function of various cell types in the ischemic brain [7]. Ischemic stroke is similarly associated with the activation of tissue plasminogen activator, a serine protease, and the generation of thrombomodulin, an anticoagulant [8]. Furthermore, the structure and function of the vascular basement membrane/extracellular matrix [9], [10], and the expression levels of aquaporin (AQP) water channels in astrocytes and endothelial cells [11], are altered during ischemic brain injury and disease. Based on these pathophysiological changes and the identified molecular targets, many directed anti-stroke therapies are now under investigation to prevent destruction of the BBB. Of the potential molecular targets, the MMPs and the AQPs form the predominant focus of our study.

MMPs belong to a family of Zn^2+^-dependent enzymes that degrade and re-establish the extracellular matrix during normal development and growth. MMPs are also implicated in BBB permeabilization and destruction [12]. During ischemic stroke, oxygen deficiency secondary to the obstruction of blood flow precipitates the inflammatory response and the upregulation of MMPs, also termed gelatinases. MMPs/gelatinases can activate numerous proinflammatory agents, disrupt the BBB, and provoke encephaledema and cerebral hemorrhage [13]. Among the MMPs, MMP2 plays an especially important role in sustaining the water balance of the brain and is commonly present on the foot processes of astrocytes and in the extracellular matrix of vascular endothelial cells.

AQPs are mostly present on brain vessels and astrocytic endfeet around lateral ventricle and ependymal cells. AQPs (AQP4 and AQP9), like MMPs, critically participate in the destruction of the BBB after CIRI, and AQP4 deficiency reportedly attenuates acute ischemic injury by reducing brain edema in acute focal cerebral ischemia [14]. Interestingly, MMPs and AQP4 expression levels seem to be co-regulated in the modulation of water homeostasis. For example, when MMPs were upregulated during CIRI-related inflammatory processes, the concomitant overexpression of AQP4 in astrocyte endfeet “OAPs” (orthogonal arrays of particles) led to particle disruption, BBB destruction, and ensuing brain edema [15].

The function of QAP-9 is very similar to QAP-4. They are both observed in astrocytes and vascular endothelial cells after stroke, and the expression of the two proteins coincides with the accumulation of water and other small solutes [16], [17]. These observations suggest that AQP-4 and AQP-9 cooperatively induce brain edema by acting as conduits to accelerate water transport between fluid compartments across the BBB and into the brain.

So far, only one drug has been approved for the treatment of ischemic stroke: the aforementioned tissue plasminogen activator, a thrombolytic agent. Unfortunately, the use of thrombolytic agents is controversial due to the increased risk of hemorrhage and neuronal death, prompting a search for safer and more efficacious ways to manage ischemic stroke. To this end, recent evidence suggests that acupuncture could be useful for the management of CIRI. Acupuncture is an alternative therapy that complements conventional medicine. Acupuncture was derived at least 2,500 years ago as an ancient Chinese treatment for illness, pain, and metabolic and pathological brain diseases. Many acupoints can be chosen for therapy, including Renzhong (governing vessel-6, GV6), Baihui (GV20), Zusanli (stomach-36, ST36), and Waiguan (triple energizer-5, TE5). Electrical stimulation in the form of electroacupuncture is usually applied to one or more acupoints for the treatment of ischemic stroke in China, enhancing the therapeutic effects of acupuncture alone. Acupuncture has recently been used to accelerate the rehabilitation of patients with brain ischemia, but its mechanism(s) of action and the added benefit of electrical stimulation for stroke remain unclear.

Our previous work indicated that electroacupuncture at GV20 and ST36 can improve neurological outcomes in CIRI model rats, and also reduce inflammation and MMP9 expression in the brain [18], [19]. The current study explored the hypothesis that acupuncture alone and electroacupuncture at GV20 and ST36 can similarly confer neuroprotection in a rat model of middle cerebral artery occlusion (MCAO) via attenuation of MMP2/AQP4/AQP9 expression and inflammatory cell infiltration in the ischemic brain. As a standard treatment for CIRI, the effects of edaravone, a free radical scavenger [20], were compared with those of acupuncture and electroacupuncture.

## Materials and Methods

### Ethics statement

All procedures were performed in accordance with the Ethics Committees of Peking Union Medical College Hospital (PUMCH, Beijing, China) and the Chinese Academy of Medical Sciences (Beijing, China), as well as the Guide for the Care and Use of Laboratory Animals (National Institutes of Health, Bethesda, MD, USA). All efforts were made to minimize animal suffering and the number of animals employed. In addition, the Ethics Committees of PUMCH and the Chinese Academy of Medical Sciences specifically approved this study (permit No. D-002).

### Rat model of CIRI

Adult male Sprague-Dawley rats (n = 80) weighing 230–250 g were housed in an environmentally controlled room at PUMCH (22±2°C with a 12 h/12 h light/dark cycle). The rats were supplied with standard rat chow and water in ad libitum. Focal cerebral ischemia was induced by MCAO, as described previously [21] with slight modifications. Briefly, MCAO was performed using an occluding suture (diameter, 0.26 mm) for 2 h, as follows. Rats were anesthetized with 10% chloral hydrate (1001g/0.3 ml) via intraperitoneal injection. The cutaneous operational area was cleaned regularly, and a 2–3 cm incision was made in the skin of the neck. The right common carotid artery, the external carotid artery, and the internal carotid artery were isolated. The external carotid artery was ligated, blood flow was blocked, and a 4–0 monofilament with a blunted tip coated with poly-L-lysine was inserted into the internal carotid artery through the external carotid artery. The suture was advanced approximately 18–20 mm beyond the carotid artery bifurcation until the origin of the middle carotid artery was blocked. After 2 h of MCAO-evoked ischemia, the suture was slowly drawn back to allow reperfusion. Rectal temperature was maintained throughout the procedure at 37±0.5°C with a temperature-regulated heating pad.

### Rat groups and treatments

Rats were randomly divided into the following five groups (n = 16 rats per group): (1) sham group S, (2) MCAO model group M, (3) acupuncture group A, (4) electroacupuncture group EA, and (5) edaravone group ED. Rats in model group M received MCAO for 2 h, followed by reperfusion for 24 h. Rats in sham group S received the same surgical procedures as those in model group M, but the suture was not advanced beyond the internal carotid bifurcation. No other treatments were given in sham group S and model group M.

Animals in treatment groups A, EA, and ED received the first treatment (acupuncture, electroacupuncture, or administration of edaravone, a free radical scavenger, respectively) after MCAO for 2 h. They were then given the second acupuncture, electroacupuncture, or edaravone treatment after 22 h of reperfusion and were euthanized 2 h later, for a total reperfusion time of 24 h in all five rat groups.

The rats in group A were given acupuncture therapy for 20 min at GV20 and left ST36 with disposable, sterile acupuncture needles (diameter, 0.32 mm; length 25 mm; Tianjin Huahong Medical Company, Tianjin, China). The needle was twisted 180 degrees at a rate of 100±5 twists per min for 1 min, with the twisting procedure repeated at 10-min intervals. Rats in group EA received acupuncture therapy at GV20 and left ST36 with disposable, sterile acupuncture needles and two electrodes (Changzhou Wujin Great Wall Medical Instrument Co., Ltd., Changzou, China), with the electrode handles conjoint on GV20 and left ST36. The rats then underwent simultaneous acupuncture and continuous-wave stimulation at a frequency of 2 Hz (intensity, 1 mA) for 20 min.

Finally, the rats in the ED group received 0.35 mg/kg of edaravone (Nanjing Simcere Dongyuan Pharmaceutical Co., Ltd., Nanjing, China) by intraperitoneal injection.

### Evaluation of neurological function

At 24 h post-reperfusion, neurological function was assessed in eight animals (n = 8 per group) by an observer who was blinded to the experimental conditions. The evaluation of neurological function was performed using the Modified Neurological Severity Score (mNSS) testing approach [22]. The mNSS is composed of a series of motor tests (i.e., muscle status and abnormal movements), sensory tests (i.e., visual, tactile, and proprioceptive assessment), balance tests, and reflex tests, and is graded on a composite scale of 0–18. The higher the mNSS, the more severe the injury.

### Assessment of infarct volume

After assessment of neurological function, rats (n = 3 per group) were narcotized by an intraperitoneal injection of 10% chloral hydrate (100 g/0.3 ml) and sacrificed by decapitation at 24 h after reperfusion. Brains were quickly removed and chilled at −20°C for 10 min. Five consecutive 2-mm coronal sections were then prepared, beginning from the anterior pole. The sections were immediately immersed in 0.1% 2, 3, 5-triphenyltetrazolium chloride (TTC; Sigma, St. Louis, MO, USA) prepared in phosphate buffered saline for 30 min at 37°C and fixed in 4% paraformaldehyde for 1 h. The TTC-stained sections were photographed, and the infarct area was measured by an observer blinded to the experimental conditions using image analysis software (ImageJ, National Institutes of Health). To account for edema and differential shrinkage resulting from tissue processing, the infarct volume percentage was calculated as: [(V_C_–V_L_)/V_C_] ×100%, where V_C_ is the volume of the control contralateral hemisphere, and V_L_ is the volume of the non-infarcted tissue in the lesioned ipsilateral hemisphere [23].

### Hematoxylin-eosin (H&E) staining

H&E histology was conducted at 24 h after reperfusion. Rats (n = 3 per group) were narcotized by an intraperitoneal injection of 10% chloral hydrate (100 g/0.3 ml) and then perfused transcardially with saline (250 ml), followed by 4% paraformaldehyde (250 ml). Brains were removed and fixed in 4% buffered paraformaldehyde at 4°C for 72 h, and then dehydrated and embedded in paraffin blocks. Coronal sections backward from the optic chiasma were cut at a thickness of 3 µm. The sections were deparaffinized and hydrated with decreasing concentrations of alcohol, stained with H&E, and photographed under a Leica DM400 microscope (Leica, Wetzlar, Germany).

### Quantitative real-time polymerase chain reaction (qPCR)

Expression levels of MMP2, AQP4, and AQP9 mRNA were determined by qPCR. Total RNA was extracted from the lesion boundary zone of the ipsilateral hemisphere (n = 3 rats per group) using an RNeasy Mini Kit (Omega Bio-Tek, Norcross, GA, USA). Extracted total RNA was then reverse transcribed to generate cDNA. The reverse transcription reaction was amplified using a Bio-Rad CFX96 Detection System (Bio-Rad, Hercules, CA, USA) with the Plexor™ One-Step qRT-PCR System (Promega, Madison, WI, USA). For MMP2, AQP4, and AQP9 amplification, the following primers were used: MMP2 forward, 5′-ACGCTGATGGCGAGTACTGCA-3′, and reverse, 5′-CCATGGTAAACAAGGCTTCGTG-3′; AQP4 forward, 5′-TCCTTTGGCCCTGCAGTTATC-3′, and reverse, 5′-AGGCTTCCTTTAGGCGACGTT-3′, and AQP9 forward, 5′-GGGTCCTATGATTGGTGCTTTC-3′, and reverse, 5′-CCCAGGATACTAACCACGAAAG-3′. The fold change in relative mRNA expression was determined using the 2^−ΔΔCt^ method [24] and glyceraldehyde 3-phosphate dehydrogenase (GADPH) as an internal control. For GAPDH amplification, the following primers were used: GADPH forward, 5′-CACAGCAAGTTCAACGGCACAG-3′, and reverse, 5′-GACGCCAGTAGACTCCACGACA-3′.

### Immunofluorescence analysis

Brain tissue sections were prepared as described above for H&E staining (n = 6 rats per group). The sections were incubated with 3% hydrogen peroxide and 5% normal goat serum at room temperature for 30 min each, followed by incubation overnight at 4°C with mouse monoclonal anti-MMP2 antibody (diluted 1∶150; Novus Biologicals, Littleton, CO, USA), mouse polyclonal anti-AQP4 antibody (diluted 1∶50; Santa Cruz Biotechnology, Santa Cruz, CA, USA), rabbit polyclonal anti-glial fibrillary acidic (GFAP) antibody (diluted 1∶500; Dako, Glostrup, Denmark), and rabbit polyclonal anti-CD34 antibody (diluted 1∶200; Abcam, Cambridge, UK). The remaining procedures conformed to standard protocols. Images were captured under a Leica Axio Observer A1 fluorescence microscope.

### Immunohistochemical analysis

Brain tissue sections were prepared as described above for H&E staining (n = 6 rats per group) and incubated overnight at 4°C with rabbit polyclonal anti-NeuN antibody (diluted 1∶300; Millipore, Billerica, MA, USA), mouse monoclonal anti-MMP2 antibody (diluted 1∶150; Novus Biologicals), rabbit polyclonal anti-AQP4 antibody (diluted 1∶150; Abcam), rabbit polyclonal anti-AQP9 antibody (diluted 1∶50; Santa Cruz Biotechnology), goat polyclonal anti-MPO antibody (diluted 1∶50; Santa Cruz Biotechnology), and mouse polyclonal anti-CD68 antibody (diluted 1∶200; Abcam). The remaining procedures conformed to standard protocols. Brain sections were photographed under a Leica DM400 microscope and analyzed using Image J software for the semi-quantitative evaluation of the integrated optical density of MMP-2/AQP4/AQP9 labeling in the ischemic penumbra and the ischemic core zone. In addition, the numbers of MPO^+^ cells and CD68^+^ cells were quantified in each 1-mm^2^ area of the ischemic penumbra and the ischemic core zone.

### Western blot analysis

The lesion boundary zone of the ischemic hemisphere (n = 4 rats per group) was dissected and homogenized in Radio-Immunoprecipitation Assay (RIPA) lysis buffer (Beyotime Biotechnology, Jiangsu, China), and total protein was separated by centrifugation at 13,000×g for 15 min at 4°C. Supernatants were harvested, and the protein concentration of each sample was determined using the bicinchoninic acid assay (Beyotime Biotechnology). For gel electrophoresis, samples were separated in 12% sodium dodecyl sulfate-polyacrylamide gels, and separated proteins were electrotransferred to polyvinylidene fluoride membranes (Millipore).

After transfer, the membranes were blocked for 2 h with 5% nonfat milk (BD-Becton, Dickinson and Company, San Antonio, TX, USA) and incubated overnight at 4°C with the following primary antibodies: mouse monoclonal anti-MMP2 antibody (diluted 1∶500; Novus Biologicals), rabbit polyclonal anti-AQP4 antibody (diluted 1∶2000; Abcam), and rabbit polyclonal anti-AQP9 antibody (diluted 1∶300; Santa Cruz Biotechnology). Blots were also incubated with a primary antibody against β-actin (Santa Cruz Biotechnology), which was employed as an internal control for the normalization of protein loading. After three washes, the membranes were incubated for 2 h with species-specific horseradish peroxidase-conjugated secondary antibodies (diluted 1∶5000; Jackson ImmunoResearch, Inc., West Grove, PA, USA). The immunoreactive protein bands were detected using an enhanced chemiluminescence kit (Millipore) and quantified using Labworks 4.6 image analysis software (UVP, LLC, Upland, CA, USA).

### Statistical analysis

All quantitative data are expressed as the mean ± the standard deviation (SD) and were analyzed using SPSS (Statistical Package for the Social Sciences) 17.0 statistical analysis software (IBM, Chicago, IL, USA). The data were subjected to a one-way analysis of variance between different groups, followed by the least significant difference t-test. In all cases, P<0.05 was considered statistically significant. All final results were analyzed by observers blinded to the experimental conditions.

## Results

### Assessment of infarct volume in CIRI model rats

**Figure 1:**
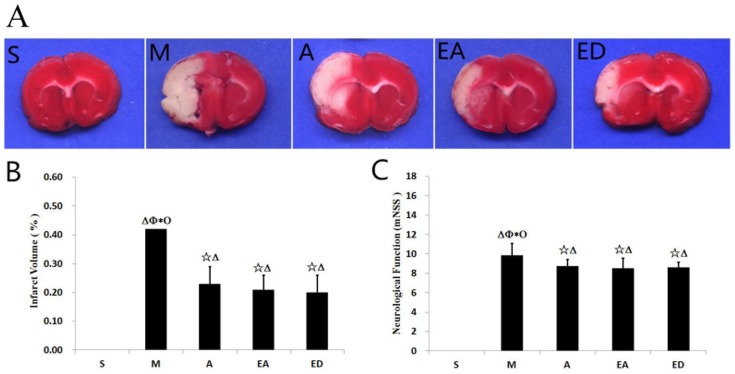
Evaluation of infarct volume and neurological scores in CIRI model rats. (A) TTC-stained brain sections showing the ischemic region (white) and the infarct area (red). (B) Infarct volume in the five experimental groups. (C) Neurological outcomes assessed by the mNSS. The three treatment groups (A, EA, and ED) demonstrated significant improvements in neurological function compared with MCAO model group M. Quantitative data (n = 8 animals per group) are given as the mean ± the SD. ^△^P<0.05 vs. sham group S; ^☆^P<0.05 vs. MCAO model group M; *P<0.05 vs. acupuncture group A; ^Φ^P<0.05 vs. electroacupuncture group EA; ^Ο^P<0.05 vs. edaravone group ED.

Infarct volume as a measure of stroke severity was first determined in the five rat groups (sham group S, MCAO model group M, acupuncture group A, electroacupuncture group EA, and edaravone group ED). Acupuncture and electroacupuncture both significantly decreased the volume of the MCAO-evoked infarct region (compare groups A and E with model group M), as assessed by TTC staining (Figure 1A and 1B). No significant differences in infarct volume were detected between the three treatment groups (A, EA, and ED), confirming the neuroprotective effect of acupuncture and electroacupuncture against CIRI.

### Neurological outcomes

To investigate whether acupuncture or electroacupuncture can influence neurological function in CIRI model rats, neurological testing was performed using the mNSS approach at 24 h after reperfusion. Rats receiving acupuncture or electroacupuncture showed significant improvements in neurological function compared with MCAO model group M (Figure 1C). No significant differences in the mNSS were found between treatment groups A, EA, and ED.

### Evaluation of cerebral histology by H&E and NeuN staining

**Figure 2:**
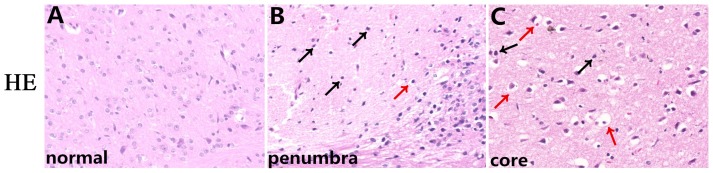
Evaluation of histopathological changes and neuronal damage in the ischemic penumbra and the core zone by H&E and NeuN staining. (A, B, C) H&E staining showing gross histopathological changes in normal, ischemic penumbra and the ischemic core at 24 h after reperfusion. The black arrows in (B, C) indicate significant nuclear shrinkage, and the red arrows indicate neuronal vacuolization. Scale bar = 50 µm.

**Figure 3:**
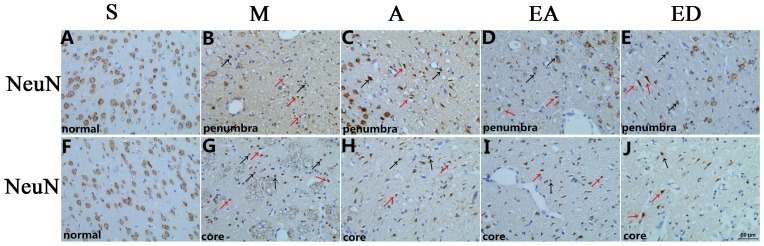
Evaluation of neuronal loss in the ischemic penumbra and the core zone by NeuN staining. NeuN immunohistochemical staining showing neuronal nuclei changes in the ischemic penumbra and the core zone at 24(black arrows) and neuronal vacuolization (red arrows) in group M. Scale bar = 50 µm.

H&E staining (Figure 2) was performed to evaluate histopathological alterations after focal ischemia, and immunohistochemical staining for NeuN, a neuronal nuclear antigen (Figure 3), was performed to evaluate neuronal loss, nuclear shrinkage, and neuronal vacuolization in CIRI model rats. Normal neurons in the contralateral hemisphere had round, lightly stained nuclei (Figure 2A), whereas dying neurons in the ipsilateral hemisphere had pyknotic nuclei (black arrows, Figure 2B and 2C) and already showed signs of vacuolization (red arrows, Figure 2B and 2C) in the core ischemic zone and the ischemic penumbra at 24 h after reperfusion. Furthermore, NeuN staining revealed a remarkable loss of neurons in MCAO model group M compared with sham group S and treatment groups A, EA, and ED, whereas the changes in the number of neurons observed between groups A, EA, and ED were not apparent (Figure 3).

### Effects of acupuncture and electroacupuncture on the mRNA expression levels of MMP2, AQP4, and AQP9

**Figure 4:**
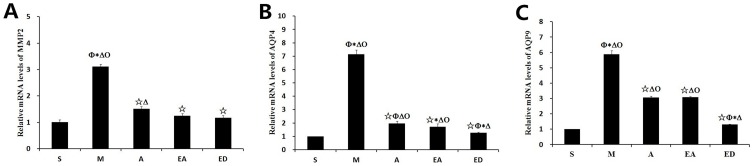
Effects of acupuncture and electroacupuncture on mRNA expression levels of MMP2, AQP4, and AQP9. Analysis of MMP2, APQ4, and APQ9 mRNA expression levels by qPCR. GAPDH was used as an internal control. Lower mRNA expression levels of MMP2, AQP4, and AQP9 were exhibited in group S vs. groups M, A, EA, and ED. The mRNA expression levels of all three target proteins were significantly lower in groups A and EA than in group M. Quantitative data (n = 3) are given as the mean ± the SD. ^△^P<0.05 vs. group S; ^☆^P<0.05 vs. group M; *P<0.05 vs. group A; ^Φ^P<0.05 vs. group EA; ^Ο^P<0.05 vs. group ED.

The mechanistic actions of acupuncture and electroacupuncture were explored by investigating their influence on MMP2, APQ4, and AQP9 mRNA expression. The mRNA expression levels of all three target proteins were significantly increased in model group M relative to sham group S, and in model group M relative to each of the three treatment groups (Figure 4). Groups A, EA, and ED showed no significant inter-group differences between the mRNA expression levels of MMP2, but the mRNA expression levels of AQP4 and AQP9 were significantly lower in edaravone group ED than in groups A and EA.

### Colocalization of MMP2 and AQP4 with GFAP and CD34 in the ischemic penumbra

**Figure 5:**
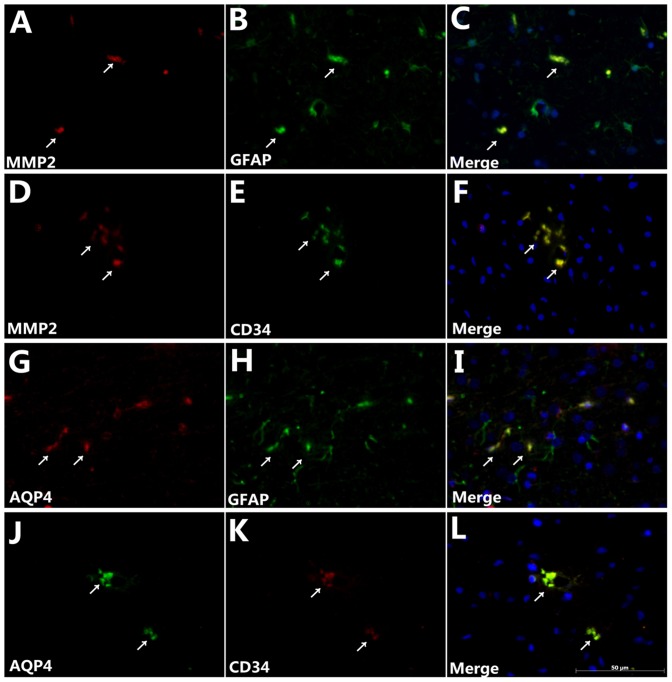
Colocalization of MMP2 and AQP4 with GFAP and CD34 in the ischemic penumbra. Immunofluorescence labeling for the MMP2, AQP4, GFAP, and CD34 expression levels in the ischemic hemisphere of MCAO model group M rats. MMP2 (red, A) and GFAP (green, B) staining and the MMP2/GFAP merge (C) show MMP2 expression on astrocyte endfeet; MMP2 (red, D) and CD34 (green, E) staining and the MMP2/CD34 merge (F) show MMP2 expression on endothelial cells; AQP4 (red, G) and GFAP (green, H) staining and the AQP4/GFAP merge (I) show AQP4 expression on astrocyte endfeet; and AQP4 (red, J) and CD34 (green, K) staining and the AQP4/CD34 merge (L) show AQP4 expression on endothelial cells. The yellow staining (arrows) in panels (C), (F), (I) and (L) indicates the colocalization of both antigens in the merged images. Scale bar = 50 µm.

To evaluate the astrocytic and vascular localization of MMP2 and AQP4 in the ischemic penumbra of CIRI model rats, immunofluorescence analysis was employed using specific primary antibodies against MMP2, AQP4, GFAP (to label astrocytes), and CD34 (to label the endothelial cell components of blood vessels). Figure 5 shows colocalization of MMP2 and GFAP (Figure 5A–5C), MMP2 and CD34 (Figure 5D–5F), AQP4 and GFAP (Figure 5G–5I), and AQP4 and CD34 (Figure 5J–5L) in MCAO model group M. These results indicate that AQP4 and MMP2 were both expressed in astrocyte endfeet and endothelial cell-derived blood vessels within the infarct area.

### MMP2, AQP4, and AQP9 protein expression in the ischemic penumbra and the core zone

**Figure 6:**
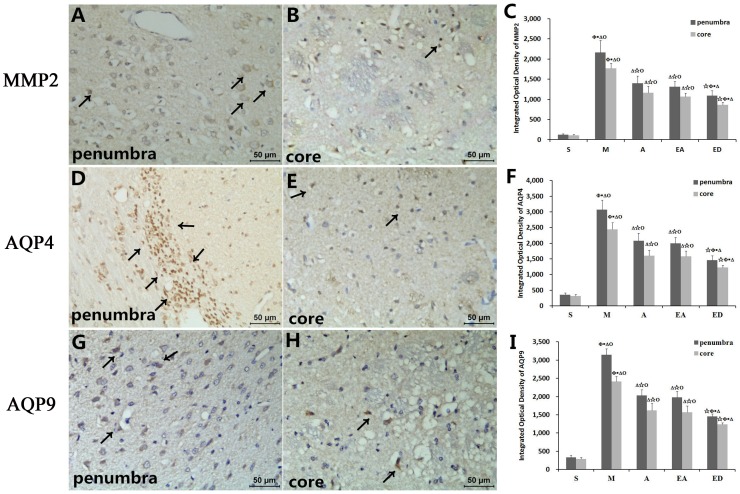
Protein expression of MMP2, AQP4, and AQP9 in the ischemic penumbra and the core zone. MMP2, AQP4, and AQP9 immunoreactivity (A, B, D, E, G, and H) and integrated optical density of MMP2/AQP4/AQP9 labeling (C, F, and I). Quantitative data (n = 6) are given as the mean ± the SD. ^△^P<0.05 vs. group S; ^☆^P<0.05 vs. group M; *P<0.05 vs. group A; ^Φ^P<0.05 vs. group EA; ^Ο^P<0.05 vs. group ED. Arrows indicate the immunoreactive area for each target protein. Scale bar in (A) = 50 µm.

Next, we explored the hypothesis that acupuncture and electroacupuncture can regulate MMP2, AQP4, and AQP9 expression in the ischemic penumbra and the core zone by performing an immunohistochemical analysis of target protein expression, followed by semi-quantitative measurement of the integrated optical density of MMP2/AQP4/APQ9 labeling. Consequently, MMP2, AQP4, and AQP9 expression levels were higher in the ischemic penumbra than in the ischemic core for all five groups of rats (Figure 6A, 6B, 6D, 6E, 6G, and 6H). However, significantly lower amounts of each protein were detected in group S vs. groups M, A, EA, and ED, and in groups A, EA, and ED vs. group M. No statistically significant differences were discerned between groups A and EA (Figure 6C, 6F, and 6I).

### Protein expression levels of MMP2, AQP4, and AQP9 in the lesion boundary zone of the ipsilateral hemisphere

**Figure 7:**
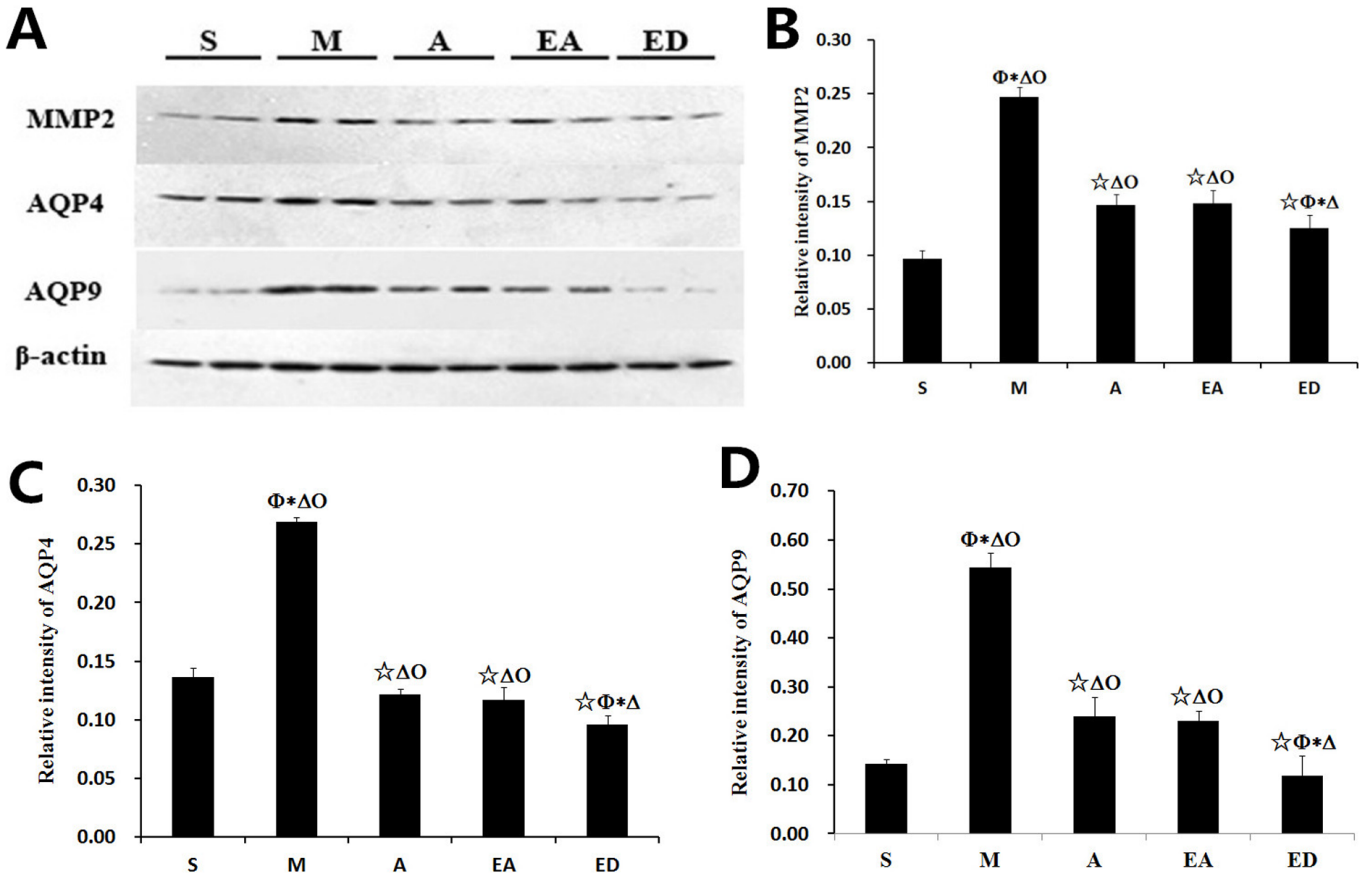
Western blot analysis of MMP2, AQP4, and AQP9 protein expression in the lesion boundary zone of the ipsilateral hemisphere. (A) Western blot analysis of MMP2, AQP4, and APQ9. (B–D) Relative intensity of MMP2 (B), APQ4 (C), and AQP9 (D) in each of the five rat groups. Relative intensity is defined as the intensity of the target protein normalized to that of β-actin. Quantitative data (n = 4) are given as the mean ± the SD. ^△^P<0.05 vs. group S; ^☆^P<0.05 vs. group M; *P<0.05 vs. group A; ^Φ^P<0.05 vs. group EA; ^Ο^P<0.05 vs. group ED.

To further investigate the influence of acupuncture and electroacupuncture on the expression patterns of MMP2, AQP4, and AQP9, Western blotting analysis was used to compare target protein levels in the lesion boundary zone in the five rat groups. The Western blotting data (Figure 7) verified the immunohistochemical findings (Figure 6), in that the protein expression levels of MMP2, AQP4, and AQP9 were all significantly higher in group M than in the other four groups (P<0.05). All three proteins were expressed at similar levels in groups A and EA, while the lowest protein expression levels were found in group ED (Figure 7).

### Quantification of MPO^+^ and CD68^+^ cells in the ischemic penumbra and the core zone

**Figure 8:**
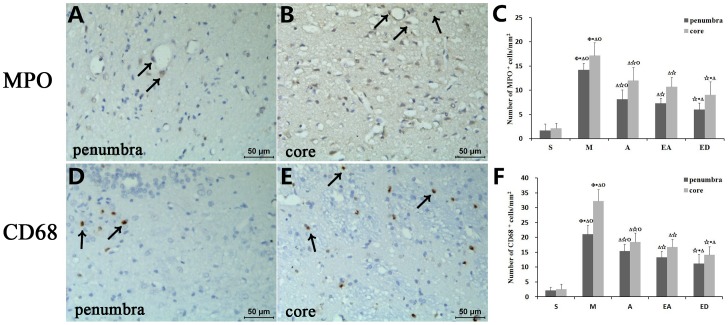
Quantification of MPO^+^ and CD68^+^ cells in the ischemic penumbra and the core zone. Representative photomicrographs showing immunohistochemical staining for MPO and CD68 in the ischemic penumbra (A, D) and the core zone (B, E) at 24 h after reperfusion. Brown spots indicate MPO^+^ cells, the majority of which were present within the ischemic core vs. the ischemic penumbra for all five rat groups. (B, E) The majority of CD68^+^ cells were also present in the core zone. (C, F) Bar graphs showing the quantification of MPO^+^ and CD68^+^ cells. The numbers of MPO^+^ and CD68^+^ cells were significantly higher in both the ischemic penumbra and the core zone for group M vs. the other four groups, whereas no significant differences were found between groups A and EA. Quantitative data (n = 6) are given as the mean ± the SD. ^△^P<0.05 vs. group S; ^☆^P<0.05 vs. group M; *P<0.05 vs. group A; ^Φ^P<0.05 vs. group EA; ^Ο^P<0.05 vs. group ED. Arrows in (A), (B), (D), and (E) show the MPO^+^ and CD68^+^ immunoreactive cells. Scale bar = 50 µm.

Finally, immunohistochemical staining for MPO and CD68 was performed in the ischemic hemisphere, followed by quantification of MPO^+^ and CD8^+^ cells in the ischemic penumbra and the core (Figure 8C and 8F). Immunostaining for both antigens was more intense in the ischemic core than in the penumbra for all five rat groups (Figure 8A, 8B, 8D, and 8E). Likewise, cell counting revealed that the majority of MPO^+^ and CD68^+^ cells, indicative of neutrophils and monocytes/macrophages, respectively, were present within the ischemic core. Significantly higher numbers of MPO^+^ and CD68^+^ cells were found in both regions for group M vs. the other four groups. But no significant differences were detected between groups A and EA (Figure 8C and 8F). However, the ED group showed the lowest number of infiltrating MPO^+^ and CD68^+^ cells relative to A, EA treatment groups (Figure 8C and 8F).

## Discussion

Acupuncture and electroacupuncture are both potential therapeutic strategies to repair brain injury and improve functional outcomes following acute ischemic stroke. Here, we showed that acupuncture or electroacupuncture at Baihui (GV20) and left Zusanli (ST36) significantly reduced the infiltration of inflammatory cells and the expression of the proinflammatory enzyme, MMP2, in CIRI model rats. Acupuncture and electroacupuncture also significantly attenuated the expression of the water channel proteins, AQP4 and AQP9, in the ischemic brain, suggesting that the protective mechanisms of these alternative treatments are partially dependent on the mitigation of inflammation-related brain edema. Consistent with the smaller observed infarct size, acupuncture and electroacupuncture both promoted significant improvements in the mNSS in CIRI model rats, indicative of enhanced neurological function.

Cerebral ischemia and edema are frequently associated with neuroinflammation, augmented production of proinflammatory mediators, and infiltration of inflammatory cells (e.g., MPO^+^ neutrophils and CD68^+^ monocyte/macrophages). The majority of the infiltrating MPO^+^ and CD68^+^ cells observed in this investigation were present within the core zone of the infarct area after CIRI, rather than in the peri-infarct penumbra. These cells were particularly prominent in model group M relative to the other four experimental groups and in A and EA groups relative to both S group. However, treatment groups A and EA showed no significant differences between the number of infiltrating MPO^+^ and CD68^+^ cells, suggesting that acupuncture and electroacupuncture are equally effective in their amelioration of certain aspects of inflammatory brain injury.

The BBB is critically involved in the maintenance of brain homeostasis. In the case of CIRI, injured brain cells (microvessel/capillary endothelial cells, pericytes, astrocytes, neurons, and others) and extracellular matter (basement membrane/extracellular matrix and tight junctions) increase the vascular permeability of the BBB and the uptake of abnormal quantities of water, thereby evoking secondary brain damage. Under normal conditions, total cessation of blood flow does not provoke dysfunction of water homeostasis, suggesting that some residual blood flow is required to develop brain edema [25]. Obviously, reperfusion worsens capillary vascular damage and augments ischemic cerebral edema. [26]. The detrimental impact of these assorted events on BBB integrity directly results in brain edema after stroke.

MMPs and other proteases are products of a molecular inflammatory cascade that damages the BBB following CIRI. BBB tight junction proteins and basal lamina proteins,which form the endothelial barrier, are vulnerable to attack by MMPs. MMP2 is especially important in astrocytes and endothelial cells, and its activation generally leads to a biphasic (transient and 24–48 h in duration) opening of the BBB [27]. For this reason, agents that protect the brain from edema by interfering with BBB are under pursuit by many researchers. Notably, our study showed that MMP2 colocalized with both GFAP in astrocytes and CD34 in endothelial cells within the ischemic penumbra, strengthening the idea that synthetic MMPs inhibitors could be used in CIRI management [28], [29]. We also showed that acupuncture and electroacupuncture significantly reversed MMP2 upregulation in CIRI model rats, although edaravone was more effective than either acupuncture or electroacupuncture.

AQPs, like MMP2, play essential roles in the pathogenesis of brain edema. The MCAO model, which utilizes focal brain ischemia to impart tissue damage, was previously reported to increase AQP4 expression in the infarct zone shortly after the insult [30]. On the other hand, another study suggested that AQP4 inhibition may provide a new therapeutic option for reducing brain edema [31]. Yet another study reported two peaks of brain swelling following CIRI, coinciding with two peaks of AQP4 expression in both the infarct and the peri-infarct area [32], [33].

AQP9, along with AQP4, is associated with brain damage after CIRI. AQP9 is upregulated in the ischemic core and the lesion border [33], with lower expression reported in the core [34]. Our data showed elevated expression of APQ9 and AQP4 in both the ischemic penumbra and the core, but the highest expression was found in the core zone. We additionally demonstrated that acupuncture, electroacupuncture, and edaravone significantly downregulated AQP4 and AQP9 mRNA and protein levels after CIRI, again implying that AQP4 and AQP9 work together to cause brain edema.

Owing to the complex and multifaceted nature of cerebral ischemic stroke, sequential staging of therapy by a combination of different mechanistic approaches will probably prove most useful in stroke management. Nowadays, acupuncture has gained increasing popularity in modern health care, garnering new support from a myriad of scientific investigators. The popular use of acupuncture has also inspired many scientists to explore ancient traditional medical technology alongside conventional medicine. Although the precise manner in which acupuncture functions is unknown and requires further study, many researchers now claim that acupuncture and electroacupuncture are beneficial for treating ischemic stroke [35]. Indeed, the assorted effects of acupuncture and electroacupuncture on the generation and blockade of free radicals, intracellular calcium, inflammation-related cytokines, and/or vasogenic edema have all been proposed to explain their possible mechanisms of therapeutic action [36], [37], [38], [39], [40].

The list of acupoints affecting the various meridians of the body is quite long, but the Baihui (GV20), Zusanli (ST36), Neiguan (pericardium-6, PC6), Weizhong (bladder-40, BL40), Sanyinjiao (spleen-6, SP6), Chize (lung-5, LU5), Renzhong (governing vessel-6, GV6), and Waiguan (TE5) locations are the most frequently chosen acupoints. Special acupuncture manipulations are also clinically used to improve self-care ability and quality of life in ischemic stroke patients, including scalp acupuncture and resuscitating acupuncture [41].

Traditional Chinese Medicine theory holds that GV20 belongs to the governing vessel, which in humans is located on the top of the head at the intersection of the middle sagittal line and the connection of the two ear apexes. GV20 functions to collect the yang around the body. After stimulation of GV20, the local yang is dispersed over and energizes the entire body. ST36 is located at 3 cm below Dubi (stomach-35, ST35) and one finger's breadth before the anterior crest of the tibia, and is utilized as an acupoint for treating digestive system diseases (e.g., gastroplegia, functional dyspepsia, and intestinal obstruction). ST36 is one of several acupoints of the stomach meridian, which is rich in both Qi and blood, and thus stimulation at ST36 has the capacity to modulate the function of the entire body. Once Qi and blood are enriched, the body can again be activated. Of relevance to the current study, simultaneous stimulation of GV20 and ST60 reportedly has a synergistically beneficial effect on the attenuation of brain ischemia [42].

Electroacupuncture, which can deliver continuous stimulation to acupoints, is currently under investigation for the management of ischemic stroke in experimental animals and in clinical practice [43], [44]. Previous work compared the therapeutic efficacy of acupuncture at ST36 and GV20 vs. electroacupuncture at ST36 and GV20 in rats undergoing CIRI and suggested that both treatments significantly increased hippocampal cell proliferation relative to the control. However, electroacupuncture delivered potentially greater benefits than acupuncture alone in terms of neuroblast plasticity [45].

According to the results of our study, CIRI rats receiving acupuncture at GV20 and ST36 or electroacupuncture at GV20 and ST36 with continuous-wave stimulation had better neurological scores and reduced brain infarction volumes than CIRI rats receiving no post-operative treatment. However, no significant differences were found between acupuncture and electroacupuncture in MMP2/AQP9 expression or the numbers of MPO^+^ and CD68^+^ cells. In fact, the only significant difference between groups A and EA was a tempered expression of AQP4 mRNA in group EA. Thus, acupuncture and electroacupuncture may share the same mechanism of neuroprotective action, with the added stimulation of electroacupuncture conferring a slight therapeutic advantage.

Edaravone is a free radical scavenger and the first medication with demonstrated efficacy for cerebral ischemia [46]. Edaravone is commonly used in China and was therefore employed as a drug control for acupuncture and electroacupuncture in the present study. Overall, the ED group presented with the lowest number of infiltrating MPO^+^ and CD68^+^ cells, protein expression levels of MMP2, AQP4, and AQP9, and mRNA expression levels of AQP4 and AQP9 relative to the other four groups. The ED group showed improvements relative to the A and EA groups; therefore, it is possible that the acupuncture and electroacupuncture techniques explored herein will require further optimization for maximum therapeutic efficacy.

In conclusion, the results of the present investigation indicate that acupuncture and electroacupuncture are effective treatments for brain tissue injury and neurological deficits following CIRI in rats. Therefore, this study adds to the growing arsenal of research supporting the view that acupuncture and electroacupuncture, which are derived from Traditional Chinese Medicine, can serve as complementary and alternative treatments to supplement the conventional management of ischemic stroke.
